# Nonsurgical Treatment of Periodontitis in Menopausal Patients: A Randomized Control Trial

**DOI:** 10.1155/2024/6997142

**Published:** 2024-03-13

**Authors:** Hadir F. Eldessouky, Magda Marie

**Affiliations:** ^1^Department of Periodontics, Faculty of Dentistry, King Abdulaziz University, Jeddah, Saudi Arabia; ^2^Department of Oral Medicine and Periodontology, Faculty of Dentistry, Ain Shams University, Cairo, Egypt; ^3^Department of Oral Medicine and Periodontics, Faculty of Dentistry, Alexandria University, Bab Sharqi, Alexandria Governorate 5424041, Egypt

## Abstract

**Background:**

Menopause is typically accompanied by significant systemic and oral manifestations, including hormonal changes and increased susceptibility to periodontal disease, which may involve inflammatory biomarkers like aspartate aminotransferase (AST) and osteocalcin in gingival crevicular fluid (GCF). The study is aimed at evaluating the effectiveness of regular inoculation of polyunsaturated fatty acids (PUFAs) as an adjunctive treatment for menopausal women's periodontitis.

**Methods:**

Twenty elderly women with chronic periodontitis were split evenly into two groups by random assignment. Patients in group II (the research group) were given soft gelatin capsules containing PUFAs to be consumed directly once daily for 12 months, as opposed to group I (the control group), who received soft gelatin capsules containing some olive oil (placebo). Scaling and root planning (SRP) were used to address periodontal disease in all cases.

**Results:**

At baseline, six and twelve months after treatment, clinical indicators and AST and osteocalcin amounts in the GCF were noted. By the conclusion of the research period, all observed clinical measurements had changed significantly and improved. In addition, there had been a significant decrease in AST levels and a nonsignificant decrease in osteocalcin levels in group II compared to group I.

**Conclusions:**

Menopausal women with periodontitis who take omega-3 fatty acid supplements in addition to SRP have better oral health. Significant improvements in clinical indicators and a notable decrease in AST levels within the GCF were observed. However, further research with larger cohorts and extended duration is needed to validate these findings and explain potential mechanisms. This trial is registered with NCT06254118.

## 1. Introduction

Menopause signifies a phase marked by profound systemic and oral changes, attributed to a gradual decline in estrogen and progesterone levels [1, 2]. This hormonal shift renders women more susceptible to periodontal disease. The changes in vascular permeability may result in gingival enlargement, inflammation, and decreased resistance to bacterial plaque if progesterone levels are high. Changes in estradiol levels can affect immune function and alter the flora and ecology of the pharynx [3, 4]. Some studies indicated that a decrease in bone mineral density may contribute to the progression of periodontal disease during menopause, whereas others indicated that osteoporosis impacts the quality rather than the quantity of alveolar bone [5, 6]. In osteoporosis, calcium deficiency leads to decreased physical activity, which may be the cause of a patient's deteriorating oral hygiene [7].

The function of cytokines activates the maturation process of osteoclasts, the proliferation process of modulated bone cells, and influences the absorption procedure of both alveolar and skeletal bone [8]. In other words, the definition of periodontitis is “The specific group of microorganisms, which is causing the inflammatory disease to the supporting tissues of the teeth” [9]. This inflammatory condition is caused by the loss of a crucial equilibrium between the virulence factors produced by microorganisms and the inflammatory host response.

There is a connection between periodontal disease, systemic health, diet, and nutrition, all of which influence oral health. The progression of the preceding lesion in periodontal tissues requires the influence of proper periodontal therapy [10]. Therefore, an endeavor to maintain optimal conditions and enhance oral health necessitates the implementation of supplemental nutritional therapy, which is beneficial to the patients [11]. There are positive associations between particular nutrients, such as calcium, vitamins C and D, and periodontal health [12].

The biomolecules of polyunsaturated fatty acids (PUFAs) include the essential fatty acids which are (i) omega-3 fatty acids (omega-3FAs) and (ii) omega-6 fatty acids (omega-6FAs). The function of these fatty acids is to provide treatment for several types of diseases [13]. It was hypothesized that omega-3FAs possess potent antibacterial properties against a variety of oral pathogens [14]. The gingival tissues initiate the origination of the microcirculation which is known as gingival crevicular fluid (GCF). The marker of inflammation analyzed in GCF has been identified as a potential diagnostic tool for periodontitis activity [15]. There are several biomarkers in inflammatory mediators that are present in oral fluids that emerged as bone turnover-related molecules and collagen degradation [16]. Aspartate aminotransferase (AST) is one of the most promising indicators of tissue destruction. AST variation in GCF may serve as an indicator of site-specific periodontal disease activity [17].

Osteocalcin is produced by osteoblasts and is known as a protein required for binding calcium in bone tissues. Kunimatsu et al. [18] reported elevated levels of osteocalcin in the GCF of patients with periodontal disease. However, it is proposed that osteocalcin primarily regulates bone formation during normal tissue turnover, with GCF osteocalcin levels showing no direct correlation with the absence or presence of periodontal disease [19]. Similarly, Golub et al. [20], Bullon et al. [21], and Afacan et al. [22] reported that there was no change in the GCF osteocalcin levels for six months after treating periodontal disease, regardless of the specific type of periodontal treatment administered.

Vitamin K supports the activation of osteocalcin, a protein synthesized by bone cells. Osteocalcin, when modified with the help of vitamin K, plays a significant role in binding calcium within bones, thereby contributing to bone strength and density. Osteocalcin is currently regarded as a valid indicator of bone turnover, and its elevation in serum has been observed during specific phases of bone turnover, including osteoporosis, fracture, and repair [23]. This study is aimed at investigating the effect of systemic administration of omega-3 fatty acids in addition to SRP on clinical periodontal parameters and GCF levels of osteocalcin and AST in menopausal women.

## 2. Material and Methods

### 2.1. Ethical Approval

This project has been reviewed and approved by the Salah Salem Dental Center Research Ethics Committee (approval number: DNH135JN20).

### 2.2. Study Design and Setting

A randomized controlled trial study was conducted between 2020 and 2022 in a clinic at Alexandria Health Insurance, Salah Salem Dental Centre. This clinical trail is registered at ClinicalTrials.gov registeration ID: NCT06254118.

### 2.3. Patient Selection

Periodontal diseases comprise a spectrum of conditions, including gingivitis and periodontitis. Gingivitis primarily involves inflammation of the gums, while periodontitis extends to the supporting structures around the teeth, resulting in the loss of connective tissue attachment and bone. Key contributors to these diseases encompass bacterial infections, plaque accumulation, genetic predisposition, and systemic conditions such as diabetes or immune disorders.

The power analysis for this study was based on time-dependent changes (mean ± SD) in periodontal probing depth (PD) and clinical attachment level (CAL) using GPower version 3.1.9.7. This widely used software allows for the determination of appropriate sample sizes in experimental designs, considering factors such as effect size, significance level, and statistical power. By utilizing GPower, the study is aimed at ensuring methodological rigor and transparency in sample size determination.

A clinically significant change in PD or CAL was determined to be a reduction of at least 2 mm. The effect size was estimated based on the observed mean changes in PD and CAL, with a conservative estimate to ensure robust statistical power. A significance level (alpha) of 0.05 and a desired statistical power of 0.80 were chosen for the analysis, aligning with standard conventions in clinical research. The determined sample size of 20 participants was based on this power analysis to adequately detect statistically significant differences in periodontal parameters and biomarker levels between the intervention and control groups.

Menopausal women, aged 45 to 55 years old, of sound health and had to undergo complete periodontal treatment, were selected from the outpatient clinic at Alexandria Health Insurance. The main reason for limiting the participants to 55 years of age was to minimize confounding variables related to age-related systemic health conditions and medication use that might influence periodontal status. Before the enrolment of the patients in the study, written consent was obtained for every participant; however, it was informed consent. The complete dental history and the medical history of the patients have been obtained from the enrolled participants. Menopause was diagnosed by taking menstrual history (such as irregular periods or their cessation), and symptoms associated with menopause like hot flashes, vaginal dryness, or sleep disturbances.

### 2.4. Inclusion and Exclusion Criteria

The inclusion criteria included the presence of a minimum of six teeth in the mouth and at least three periodontal sites in different quadrants with a probing pocket depth (PPD) ≥ 5 mm. The women experiencing menopause for at least a year were included in the study. However, patients suffering from any disorder which might in any way impact the periodontal tissue (e.g., diabetes mellitus, or any immune disorders) were excluded. Exclusion criteria also included patients who were anemic, habitual smokers, regularly used mouthwash, and had taken nonsteroidal anti-inflammatory drugs (NSAIDs), supplements, or antibiotics within the last three months. Participants in any other clinical trials were also not considered for the study.

### 2.5. Study Procedure

All patients underwent complete periodontal examination but exclusive of the third molars. This evaluation involved a thorough examination of parameters related to the periodontium, including (i) plaque index (PI) [24], (ii) gingival index (GI) [25], (iii) PPD [26], and (iv) CAL [27]. The examination was conducted using a UNC-15 periodontal probe (UNC-15, Hu-Friedy, Chicago, IL, USA). PPD and CAL were measured at 6 points around each tooth mesiobuccal (MB), midbuccal (B), distobuccal (DB), mesiolingual (ML), midlingual (L), and distolingual (DL). For this study, generalized chronic periodontitis was diagnosed when there was a documented loss of clinical attachment in more than 30% of the teeth within the oral cavity. Patients were provided with detailed instructions about oral hygiene throughout the study period.

The study cohort (*n* = 20) was divided into two groups where half of the patients were assigned to the control group (group I) while the remaining half to the study group (group II). This allocation was done using a randomized allocation method. An independent researcher, not directly involved in the study, utilized a computer-generated random number sequence to assign participants to either the control group (group I) or the study group (group II). This randomization process ensured an unbiased and equitable distribution of participants across both groups, reducing the risk of selection bias and confounding variables influencing the study outcomes. Each participant was assigned a unique identifier, and randomization was executed before the commencement of the treatment phase, ensuring an even distribution of participants with similar baseline characteristics and minimizing the impact of potential confounders.

The control group was followed with the treatment of SRP which is nonsurgical root planning + scaling + oral placebo (soft gelatinous capsule containing olive oil). The SRP was administered by trained and calibrated dental professionals over multiple sessions, with each session concentrating on specific areas necessitating intervention. During the treatment sessions, periodontal curettes (Gracey Curettes, Hu-Friedy Co., Chicago, IL) and ultrasonic inserts (Mectron-Carasco GE, Italy) were utilized to remove dental plaque, calculus deposits, and bacterial biofilm from the tooth surfaces, particularly targeting areas with PPD of ≥5 mm. The completion of periodontal treatment was based on predefined clinical criteria, i.e., reduced PPD, improved gingival health, and the absence of calculus and plaque in the treated areas. The treatment duration spanned several weeks, ensuring a comprehensive and effective intervention.

The professionals conducting clinical measurements and administering periodontal treatment were kept unaware of the participants' group assignments. This double-blind approach is aimed at mitigating potential bias and uphold the integrity of the study. Additionally, the professional performing clinical measurements remained uninformed about which capsule (omega-3FAs or placebo) each patient used, ensuring unbiased assessments.

On the other hand, the study group (group II) was treated with SRP+ oral 1000 mg omega-3FA (Omega 3 Plus, SEDICO Pharmaceutical, 6th of October City, Egypt) soft gelatinous capsule composed of docosahexaenoic acid (DHA) and eicosapentaenoic acid (EPA). Participants were instructed to take 1000 mg soft gelatinous capsules of omega-3FAs containing DHA and EPA daily at the initiation of the first treatment session. The specific dosage and frequency were determined based on the study protocol. Instructions regarding the timing of intake (e.g., with meals or at a specific time of day) were provided to ensure consistency in administration across participants. Patients were asked to maintain a record or inform the researchers about any missed doses or unused capsules to monitor adherence to the treatment regimen and consider its potential impact on the study outcomes.

Two different sets of professionals were involved to maintain the integrity and objectivity of the data collection process. Both treatment regimens spanned over twelve months.

#### 2.5.1. Assessment of Clinical Parameters

The clinical parameters, including (i) plaque index (PI) [24], (ii) gingival index (GI) [25], (iii) PPD [26], and (iv) CAL [27] represent full-mouth measurements recorded after a comprehensive examination of all teeth within the oral cavity, encompassing multiple sites per tooth, using standardized clinical assessment protocols. Participants in the study underwent a structured assessment schedule involving periodic clinical evaluations and GCF sampling at specific intervals. The assessments were conducted at the following time points:

- Baseline assessment (before treatment initiation)
- Six-month follow-up assessment
- Twelve-month follow-up assessment

The sequence of clinical measurements and GCF sampling remained consistent across all follow-up visits to enable a correlation between clinical findings and biochemical analyses. During each visit, in addition to emphasizing proper oral hygiene, scaling was performed as per the study plan. However, root planning was not conducted at the 6th-month follow-up to avoid an impact on the subsequent 12th-month assessment. The standardized oral healthcare training provided to participants included comprehensive guidance on proper brushing and flossing techniques, advice on adjunctive oral hygiene aids, dietary recommendations for oral health, strategies to prevent oral problems such as smoking cessation, and the importance of regular dental check-ups.

#### 2.5.2. Collection of Gingival Crevicular Fluid (GCF)

GCF samples were collected from both single-rooted and multirooted teeth, focusing on sites with PPD of 5 mm or more, as these deeper pockets often indicate periodontal inflammation and increased GCF flow. The samples were taken from the deepest point of the pocket whether M or D. No samples were taken from the sulcus. GCF samples were obtained from specific sites within the periodontal pockets using sterile filter paper strips (Perio-paper, IDE Interstate, and Amityville, New York, United States of America). The collection technique involved inserting the filter paper strips into the crevice of the periodontal pocket with a probing depth greater than 5 mm, ensuring contact until resistance was encountered, and leaving them in place for at least 30 seconds to absorb the GCF. The collected samples were handled carefully to prevent contamination, and any filter paper contaminated with blood was discarded. The filter papers soaked in the required fluid were then placed in Eppendorf tubes which already contained 300 *μ*L phosphate buffered saline (PBS). All the collected samples were stored at the maintained temperature of -20°C for further analysis [28]. The individual samples were analyzed separately to capture localized variations in inflammatory biomarkers or biochemical components.

#### 2.5.3. Assessment of AST Level

The next step was the evaluation of AST in the collected GCF. The assessment was done using the VITROS Dry Technology 60 Chemistry System which can provide accurate results using even very small amounts of GCF (~10 *μ*L). The principle behind the test is to evaluate the enzymatic activity of AST to convert the amino group of aspartates where the oxidation process of nicotinamide adenine dinucleotide (NAD) + hydrogen (H) (NADH+). This activity is measured by the reflectance spectrophotometry at 340 nm wavelength and 37°C. The change in rate assesses the enzymatic activity as per Bergmeyer et al.'s [28] method.

#### 2.5.4. Assessment of Osteocalcin Level

The evaluation of the level of osteocalcin in the GCF sample, using enzyme-linked immunosorbent assay (ELISA) kits (Ani Biotech Oy, AvioBion, Finland), was the next step in the study. The osteocalcin levels are measured in nanograms per milliliter (ng/mL) when assessed using ELISA kits. The principle applied in the sandwich-type ELISA kit is the presence of a monoclonal osteocalcin which is adsorbed onto the well of the kit and then binds to the osteocalcin (present in the sample). The amount of osteocalcin present was assessed using standard curves based on dilutions and measured at the color intensity of 450 nm. All calibrations were done in duplicate to reduce errors [29].

### 2.6. Statistical Analysis

The statistical analysis in this study was conducted using the statistical software SPSS (Statistical Package for the Social Sciences) version 23. To assess changes over time within each group (intragroup analysis), a paired *t*-test was conducted for continuous variables, such as PI, GI, PPD, and CAL. This statistical approach allowed the examination of significant differences in these parameters at various time points within the control (group I) and intervention (group II) groups.

## 3. Results

The comparison of the PI scores between groups I and II represent no significant difference at the initial stage and the 6 months. There was a decrease in scores from baseline to 12 months in both groups, but the decrease was more significant in group II. Regarding GI, PPD, and CAL, group II showed statistically significant improvement, i.e., a decrease in the scores both at intragroup (12 months vs. baseline) as well as intergroup (group I vs. group II) levels. However, the same did not hold for the intragroup comparison for group I (Table 1). These differences highlight the greater efficacy of the intervention (group II-SRP + omega-3FAs) in improving periodontal parameters compared to the control group (group I-SRP + oral placebo).

The comparison of AST levels in group II displayed a statistically significant decrease throughout the study period, as well as when compared to group I. However, this was not observed for the AST levels in group I (Table 2). Similar to AST levels, osteocalcin levels in the GCF had a statistically significant decrease in group II during the entire study period, but not when compared to group I (Table 3).

## 4. Discussion

The epidemiology of periodontitis has been impacted by factors such as discrepancies in definition, prevalence, and severity. With increasing age being directly associated with periodontitis, the presence of menopause is now considered a major risk factor for the progression and severity [30]. Periodontal disease is a progressive disease associated with tissue destruction, as a result of the host's response to stimuli (genetic, systemic, and environmental) and bacterial antigens [31]. A wide array of available host-modulating therapies are known to impact different aspects of host immunity response by reducing the exaggerated inflammatory response which helps in wound healing and periodontal stability [32]. On the other hand, the process of scaling and plaque removal helps in bacterial reduction. Pharmacotherapy and dietary changes are considered important preventive steps for periodontitis [33].

In this study, we evaluated the use of dietary omega-3 PUFAs (1000 mg of EPA + DHA) as an adjunct to the periodontitis nonsurgical treatment in menopausal women. In the present study, group I did not experience a reduction in GI despite undergoing SRP, while, group II showed a substantial decrease in GI following the 12 months. This clarifies that the introduction of omega-3 FAs in group II improved gingival inflammation, suggesting that factors related to the anti-inflammatory effects and lipid mediator production of omega-3 FAs worked beyond the reduction of microbial burden achieved by SRP. The benefits of dietary omega-3 PUFA supplementation have been studied in other inflammatory disorders [34]. The omega-3 PUFAs help in the resolution of inflammation which occurs by the lipoxin. The lipoxins are lipid mediators that are endogenous anti-inflammatory and proresolution in nature production formed as a result of arachidonic acid metabolism by transformation circuits by lipoxygenase enzyme [35]. Several studies have reported the production of lipid mediators (pro resolvins, resolvins, and protectins) as a result of the metabolism of omega-3 FAs and are known to have anti-inflammatory and immunoregulatory effects and thus help in wound healing [34, 36, 37].

PPD and CAL did not indicate any significant reduction in group I after 6 and 12 months in this study. However, for group II, both PPD and CAL reduction had been significant and stable. When comparing the 2 groups, no difference was observed at baseline and 6-month group, but the difference was significant after 12 months which could be attributed to the beneficial effect of omega-3 both as a potent anti-inflammatory and antibacterial agent against a range of oral pathogens including Porphyromonas gingivitis which helps in the control of plaque formation on the tooth surface [38]. Omega-3 FAs may play a protective role for the asaccharolytic microbial pathogens by the deduction of the host inflammatory responses. Less tissue damage causes Porphyromonas gingivalis which provides unsustainability to microbes unable to form their protein-derived energy source [39]. Omega-3 plays an anti-inflammatory role by reducing osteoclastic activity and thus gingival inflammation leading to decreased alveolar. These anti-inflammatory effects of omega-3 have a similar mode of action as that of other host modulators such as doxycycline but without the side effects [40, 41].

According to the agreement of investors, the results which are obtained from the study are that omega-3 FAs tend to achieve level again by reducing gingival inflammation and pocket depth [36, 42]. As compared, Martinez et al. [43] reported no effect of dietary omega-3 supplementation on the clinical outcome of treatment of periodontitis which could be allocated the sample size of 10 patients and a short duration of treatment (4 months). AST is released in the extra space of the cellular membrane during tissue necrosis. Thus, the enhanced development in the levels of AST is present in the GCF of diseased sites in comparison to healthy sites. These are active sites of periodontal tissues [44]. So, it is hypothesized that during gingival inflammation, its concentration will be increased, and after the SRP, when the microbial load is reduced, AST levels in periodontitis may also be decreased [45].

In group I, after SRP when the microbial load was reduced, periodontal destruction was also reduced which resulted in the decreased AST concentrations in GCF, but the reduction was not very significant as it decreased from 1326 ± 16.35 to 821 ± 11.30 after 12 months. This might be due to residual inflammation and ulcerations in the tissues even after SRP of the deep pockets. SRP reduced the inflammation but might be unable to eliminate it in deep pockets. SRP alone is not sufficient to remove the inner lining epithelium of the pocket. The presence of inflammation is believed to be related to the penetration of antigenic substances via the gingival sulcus and junctional epithelium. Since deep periodontal pockets having larger surface areas are exposed to the external environment, a higher concentration of AST is expected [45]. This finding also supports that only SRP may not be a sufficient treatment modality for complete debridement of tooth surface and inflamed periodontal tissue; an additional approach may be required for the elimination of pockets, as remaining pocket depths are difficult to maintain by the patient. This is following Smith et al. [46]. The significant reduction in the AST levels as a result of adjunctive use of dietary omega-3 FA supplementation thus improved the outcome of standard SRP. The anti-inflammatory effect of omega-3 PUFAs via the resolvins includes the reduction of upstream proinflammatory cytokines that are known to result in the apoptosis of the neutrophils as well as the nonphlogistic (nonheat-producing or noninflammatory) recruitment of monocytes [47, 48].

Osteocalcin level measures bone formation as well as impacts bone resorption in vitro and can be considered as a marker of periodontal disease active site [20, 49–52]. The study result showed an increment in the level of GCF osteocalcin in group I in comparison to group II. This was contrary to the findings of Matouga et al. [49] who reported a reduction in the cases of chronic periodontitis after SRP. Our results did find concordance with the results of Golub et al. [20] who did not report any objectives in the level of osteocalcin after SRP in chronic periodontitis patients. On the other hand, the level of osteocalcin showed a reduction at all intervals but was statistically insignificant. GCF level of osteocalcin in periodontal disease can have variations.

Contrary to our study's findings about specific clinical periodontal parameters after SRP in Group 1 showing no improvement, several previous studies presented contrasting findings. One study highlighted the efficacy of initial periodontal therapy by demonstrating reduced neopterin levels in pre- and postmenopausal groups, suggesting neopterin as a potential diagnostic marker for periodontal disease [53]. The decreased levels of neopterin after nonsurgical periodontal treatment proposed the efficacy of nonsurgical therapy [54]. Another study observed a significant reduction in serum ferritin levels following nonsurgical periodontal treatment in postmenopausal women with chronic periodontitis, indicating systemic implications of periodontal therapy [55]. In contrast to the present study findings, a study noted insignificant changes in GCF osteoprotegerin levels postphase I therapy in periodontitis patients and suggested the minimal influence of menopause on periodontal status within their study scope [56]. Another study suggested the complex association between estrogen status and local alkaline phosphatase levels in GCF, challenging alkaline phosphatase as merely reflective of local inflammation [57].

The study group with omega-3 FA supplementation reported that there is a clear indication of the reduction in the GCF osteocalcin level when compared to the placebo group. A decrease in GCF osteocalcin levels in the postperiodontal therapy meant higher levels after bone resorption but lower levels in the periodontal healing period. These findings may be related to the omega-3 FA supplementation that reduced the inflammatory reactions and helped in periodontal healing. The group receiving dietary omega-3 PUFAs (group II) displayed a significant reduction in GCF AST levels compared to the control group (group I), suggesting a potential mitigation of tissue damage and inflammation following periodontal therapy augmented by omega-3 FAs. Moreover, group I displayed increased osteocalcin levels compared to group II indicating variations in bone formation and resorption dynamics within the periodontal environment. The observations about GCF AST and osteocalcin levels provide additional insights regarding the impacts of omega-3 FAs that have a potential impact on tissue integrity, inflammatory reactions, and the complex mechanisms governing bone remodeling throughout the process of periodontal healing. The main limitation of the study is the small sample size and relatively short follow-up duration; however, the observed outcomes emphasize the relevance of host-modulating therapies in the context of periodontitis.

## 5. Conclusion

In conclusion, the supplementation of Omega-3 FAs alongside SRP demonstrated notable associations with periodontitis in menopausal women. The study's significant reduction in GCF AST levels suggests potential mitigation of tissue damage and inflammation after periodontal therapy augmented by Omega-3 FAs. However, the study showcased differing trends in GCF osteocalcin levels, indicating a potential role of Omega-3 FAs in reducing inflammatory reactions and aiding in periodontal healing processes. These findings highlight the potential benefits of Omega-3 FAs as a dietary supplement in enhancing the management of chronic periodontitis among menopausal women, particularly in cases involving deep periodontal pockets. The study findings have recommended the development of more effective treatment options targeted at mitigating periodontal disease. It is also crucial to acknowledge that while the study suggests potential benefits of Omega-3 FAs in aiding chronic periodontitis management among menopausal women, further extensive research is necessary to establish the definitive impact and effectiveness of Omega-3 supplementation.

## Figures and Tables

**Table 1:** Mean (M) and standard deviation (SD) of the all-clinical parameters in both group I and group II.

	Group I			Group II			Group I vs. group II
	*M* ± SD	Baseline vs. 6-month *t*-test	Baseline vs. 12-month *t*-test	*M* ± SD	Baseline vs. 6-month *t*-test	Baseline vs. 12-month *t*-test	
PI							
Baseline	2.55 ± 0.37			2.43 ± 0.58			
6 months	1.46 ± 0.38	1.09		1.07 ± 0.63	1.35		0.66
12 months	0.99 ± 0.40		3.38∗	0.59 ± 0.44		3.85^∗^	0.68
GI							
Baseline	1.91 ± 0.54			1.87 ± 0.63			
6 months	1.68 ± 0.38	1.29		0.43 ± 0.37	1.02		1.78
12 months	0.81 ± 0.49		1.44	0.38 ± 0.42		3.66^∗^	3.33^∗^
PPD							
Baseline	5.11 ± 0.54			5.18 ± 0.65			
6 months	4.75 ± 0.39	1.89		4.27 ± 0.55	1.97		1.54
12 months	4.89 ± 0.63		1.48	3.33 ± 0.34		3.63^∗^	3.53^∗^
CAL							
Baseline	5.31 ± 0.55			5.29 ± 0.37			
6 months	5.27 ± 0.48	1.37		4.09 ± 0.43	1.86		1.46
12 months	5.22 ± 0.65		1.69	2.99 ± 0.43		3.31^∗^	3.38^∗^

^∗^Statistically significant at *p* ≤ 0.05.

**Table 2:** Aspartate aminotransferase in GCF in both group I and group II (U/mL).

	Group I			Group II			Group I vs. group II
	*M* ± SD	Baseline vs. 6-month *t*-test	Baseline vs. 12-month *t*-test	*M* ± SD	Baseline vs. 6-month *t*-test	Baseline vs. 12-month *t*-test	
Baseline	1326 ± 16.35			1318 ± 14.33			
6 months	989 ± 25.22	11.55		623 ± 21.84	25.51^∗^		30.47^∗^
12 months	821 ± 11.30		12.31	399 ± 22.13		36.33^∗^	39.5^∗^

^∗^Statistically significant at *p* ≤ 0.05.

**Table 3:** Osteocalcin level in GCF in both group I and group II.

	Group I			Group II			Group I vs. group II
	*M* ± SD	Baseline vs. 6-month *t*-test	Baseline vs. 12-month *t*-test	*M* ± SD	Baseline vs. 6-month *t*-test	Baseline vs. 12-month *t*-test	
Baseline	1.99 ± 0.18			1.93 ± 0.28			
6 months	1.55 ± 0.25	0.47		1.37 ± 0.11	0.68		1.36
12 months	1.69 ± 0.10		0.68	1.19 ± 0.32		0.57	1.49

^∗^Statistically significant at *p* ≤ 0.05.

## Data Availability

The data will be available for review from the corresponding author on request.

## Abbreviations

PUFAs: Polyunsaturated fatty acids
Omega-3FAs: Omega-3 fatty acids
GCF: Gingival crevicular fluid
AST: Aspartate aminotransferase
EPA: Eicosapentaenoic acid
DHA: Docosahexaenoic acid
PI: Plaque index
GI: Gingival index
PPD: Probing pocket depth
CAL: Clinical attachment level
PBS: Phosphate buffered saline.

## Additional Points

*Key Findings*. The study found that menopausal women with periodontitis who took omega-3 fatty acid (omega-3FAs) supplements in addition to scaling and root planning (SRP) showed significant improvements in clinical measurements and a significant decrease in aspartate aminotransferase (AST) levels, indicating better oral health compared to those who received a placebo.
